# Assessment Tools for the Admission of Older Adults to Inpatient Rehabilitation: A Scoping Review

**DOI:** 10.3390/jcm12030919

**Published:** 2023-01-24

**Authors:** Francesca Muscat, Liberato Camilleri, Conrad Attard, Stephen Lungaro Mifsud

**Affiliations:** 1 Department of Physiotherapy, Faculty of Health Sciences, University of Malta, MSD 2090 Msida, Malta; 2 Statistics and Operations Research, Faculty of Science, University of Malta, MSD 2080 Msida, Malta; 3 Computer Information Systems, Faculty of ICT, University of Malta, MSD 2080 Msida, Malta

**Keywords:** rehabilitation, outcome measures, rehabilitation potential, older adults, geriatric assessments, scoping review

## Abstract

(1) Objective: To identify the assessment tools and outcome measures used to assess older adults for inpatient rehabilitation. (2) Design: Scoping review. (3) Data sources: ProQuest, PEDro, PubMed, CINAHL Plus with full text (EBSCO), Cochrane Library and reference lists from included studies. (4) Review method: The inclusion of studies covering patients aged >60, focusing on rehabilitation assessments delivered in hospitals in community settings. Studies reporting on rehabilitation specifically designed for older adults—testing for at least one domain that affects rehabilitation or assessments for admission to inpatient rehabilitation—were also included. Results were described both quantitatively and narratively. (5) Results: 1404 articles were identified through selected databases and registers, and these articles underwent a filtering process intended to identify and remove any duplicates. This process reduced the number to 1186 articles. These, in turn, were screened for inclusion criteria, as a result of which 37 articles were included in the final review. The majority of assessments for geriatric rehabilitation were carried out by a multidisciplinary team. Multiple studies considered more than one domain during assessment, with a high percentage evaluating a specific outcome measure used in geriatric rehabilitation. The most common domains assessed were function, cognition and medical status—with communication, vision and pain being the least common. A total of 172 outcome measures were identified in this review, with MMSE, BI, FIM and CCI being the most frequent. (6) Conclusions: This review highlights the lack of standardised approaches in existing assessment processes. Generally, older-adult-rehabilitation assessments struggle to capture rehabilitation potential in a holistic manner. Hence, a predictive model of rehabilitation for assessing patients at the initial stages would be useful in planning a patient-specific programme aimed at maximising functional independence and, thus, quality of life.

## 1. Introduction

Over the last decade, the number of persons in Malta aged >60 has increased by almost 34,000, accounting for approximately 18.9% of the total population at the end of 2020 [1]. A similar increase in pattern has been noted globally [2,3], highlighting the broadening of the top of the age pyramid.

The increase in the older-adult population stresses the importance of improved healthcare services to detect vulnerable individuals in the community as early as possible. Due to the effects of ageing, functional decline and increasing rates of disability, older adults are at an increased risk of hospitalisation, care and admission to long-term facilities. Following an acute event that would lead to hospitalisation, older adults could benefit from an inpatient rehabilitation phase, which could eventually help them, as far as possible, to return to their previous physical function [4,5]. The provision of an inpatient rehabilitation programme would reduce healthcare costs and improve patient outcomes, as it would increase functional independence, reduce re-admission rates and length of stay and would improve quality of life [6].

Geriatric rehabilitation (GR) is an inpatient programme that applies a multidisciplinary-team approach, typically consisting of a consultant geriatrician, skilled nursing staff, a physiotherapist, an occupational therapist and a social worker. It could also include a speech therapist, psychologist, podiatrist and dietician [7]. The multidisciplinary team (MDT) seeks to adopt a rehabilitative mindset, and works with patients and their caregivers, with a view to discharging patients back into the community, offering suitable support in a safe environment [5]. However, in some cases, patients would not benefit from the same intensity of rehabilitation as others, or would even not be considered eligible for inpatient GR. The process by which clinicians assess and categorise patients for GR is based on rehabilitation potential (RP).

According to the World Health Organization (WHO) the aim of rehabilitation is to maximise functional independence and facilitate psychosocial adjustments to residual disability, to allow the patient to function successfully within the community [8,9,10]. The WHO-approved International Classification of Functioning, Disability and Health (WHO ICF) framework illustrates that functional independence is conditioned by physical ability and medical inferences, and also by other areas, including social and environmental influences. This applies to the rehabilitation of young adults, but it is arguably even more complex in the GR field [8,10,11]. Numerous studies have looked at which factors could affect rehabilitation potential and success. Deeper knowledge of this could help the development of a standardised RP assessment, to be used with patients for admission to inpatient GR.

Taking all the above into consideration, this review seeks to provide an overview of the assessments in place for admitting patients to inpatient GR, and which domains are considered for rehabilitation. This review will provide the basis for the setting up of an expert panel, which would enhance the robustness of the design and development of a prediction model for GR.

## 2. Materials and Methods

### 2.1. Information Sources and Search

A review search was conducted as the starting point for the current study. An electronic, seven-step search string was developed, with guidance from an experienced librarian (Table S1). The same search string was carried out on all databases included in the review, from inception to December 2021. The choice of having all publications included, with no cut-off in years of inclusion, is due to the fact that there has not been a consensus on what are the constituents of older people in rehabilitation, particularly in deciding the potential of the patient in using established tests. The Preferred Reporting Items for Systematic Reviews and Meta-Analyses (PRISMA) reporting guidelines were used in the development of this review, together with the method guidelines of the Cochrane Collaboration. The review covered studies available in the English language. The databases searched were ProQuest, PEDro, PubMed, CINAHL Plus with full text (EBSCO) and Cochrane Library. Table S2 offers a rationale for the chosen databases. Manual searching in reference lists of included studies was also performed. Four further searches were carried out in the following sources: Journal of Rehabilitation Medicine, Quality of Life Research, Iranian Rehabilitation Journal and Journal of Gerontology.

Searches, title and abstract screening were carried out by a single researcher (FM), whereas full-text screening and data extraction was completed by FM and reviewed by two other researchers (SLM, CA). Any divergent opinions, such as the relevance of particular studies to inpatient rehabilitation, were resolved through discussions among the research team members. A sample of excluded articles is presented in Table S4. The data obtained was then recorded on a data-extraction sheet formulated for the purpose of this study (Table S3), which included information regarding the following: the study design; location of the intervention (such as a ward or hospital); participant demographics; outcome measures and assessments used; and definitions of rehabilitation given. The form was piloted by one researcher (FM) on a sample of five papers, and then discussed with the study team (SLM, CA) to ensure that the form was adequate for the review.

### 2.2. Inclusion Criteria

This review included studies that focused on rehabilitation assessments and measures delivered in hospitals in community settings. There is a divergence of views as to what constitutes the age of an older person. Some countries consider admission to geriatric rehabilitation wards at the age of 60 years and older. This review is based on that position for the sake of completeness. Rehabilitation targeted adults aged >60 [12] suffering from frailty or multiple comorbidities developed in adult years, who required assessment to measure their RP or possible improvement and recovery. Studies that included assessment of RP and outcome measures predicting rehabilitation success were also included. Studies had to report on rehabilitation specifically designed for older adults and test for at least one domain that would affect rehabilitation, or assessments for admission to inpatient rehabilitation. Moreover, the review included studies concerning participants who lived in the community prior to assessment.

Studies that presented primary research, including randomized controlled trials, non-randomised control trials, quasi-experimental studies, pre-test/post-test studies, prospective and retrospective cohort studies, case-control studies and individual case reports were also included.

### 2.3. Exclusion Criteria

The review excluded studies using outcome measures specific to conditions such as stroke, traumatic brain injuries, intensive care, palliative and end-of-life care, amputee rehabilitation, burns, substance-abuse care and specific post-COVID-19 or cardiopulmonary rehabilitation.

Studies that did not have an age restriction or a threshold of 60 years, studies that did not focus on patient evaluation measures, and studies that incorporated assessments involving digital technology or virtual reality were eliminated from the study.

Opinion pieces, editorials and books were also omitted from this review.

### 2.4. Types of Outcomes

Outcomes of interest included the following: measures of physical function and activities of daily living (PADLs and IADLs); cognitive outcome measures; and other outcome measures that had an effect on rehabilitation provided to older adults, based on the domains stated in the WHO ICF framework [8,10].

### 2.5. Data Analysis

All the studies were uploaded on Mendeley, a reference management software, with duplicate articles being removed. The studies were screened by title and abstract to identify those which met the inclusion criteria. The full text was then analysed by author, publication year, country of publication, study type and design, participant demographics, outcome measures used and clinician assessment and results. Results were described both quantitatively and narratively, noting emergent themes and taking into account the rehabilitation definition stated in the WHO ICF framework [8,10]. A systematic review was not undertaken, due to the results obtained being too broad and diverse; this prevented a transparent framework to be produced in a precise way, as required in a systematic review. Furthermore, a meta-analysis report was not carried out, due to the heterogeneity among the study populations in the included articles.

The quality of the studies was evaluated and marked as ‘yes’ or ‘no’, noting any missing information that could affect the quality of the results presented in this review. A high number of ‘yes’ values indicated that the study had a good level of transparency in the method. A table representing a sample of bias is presented in Table S5, which offers a sample of studies that had more than one bias.

## 3. Results

### 3.1. Study Selection

**Figure 1:**
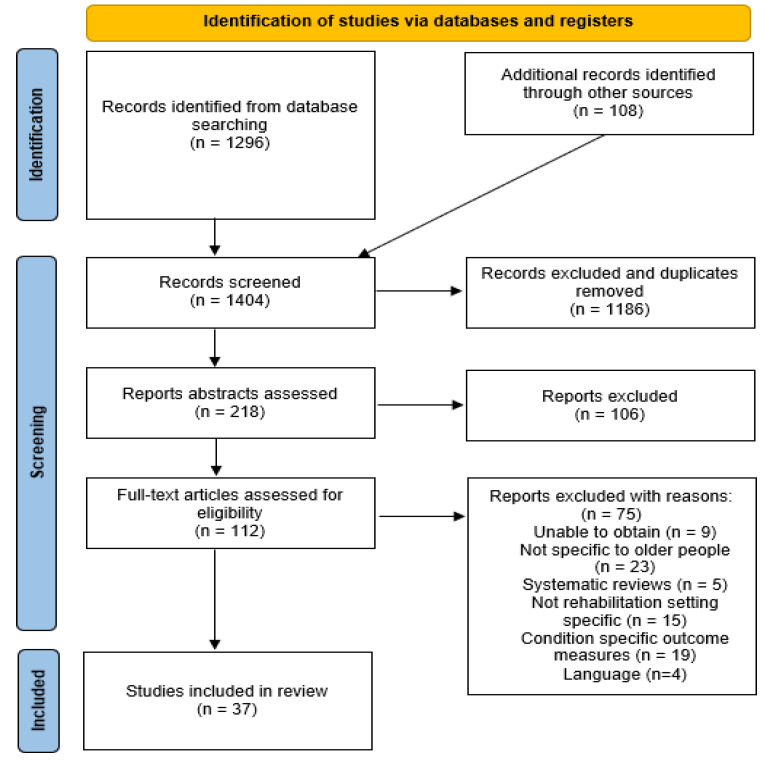
Flowchart of identification of studies via databases and registers.

In total, 1404 articles were identified through the selected databases and registers. After removing duplicates, a new total of 1186 articles was obtained, all of which were duly screened. Following inclusion-criteria screening, 37 articles were selected in the final review. This is shown in Figure 1, below; Table S17 shows the selected articles, while Table S4 presents a sample of excluded studies.

### 3.2. Study Characteristics

The 37 studies were conducted between 1969 and 2021, five of which were published during 2020—the highest number since 1969. More than half of the 37 studies were carried out in Europe (54.1%; n = 20).

The Journal of Rehabilitation Medicine numbered the highest number of articles (16.2%, n = 6), followed by the Journal of Gerontology (8.1%, n = 3) and Age & Ageing (8.1%, n = 3). The most common type was prospective longitudinal observational studies, followed by prospective cohort studies (29.7%, n = 11 and 24.3%, n = 9, respectively). The majority of the articles took place in a GR setting (73.0%, n = 27), included a sample size between 200 and 299 patients (27.0%, n = 10) and had a data-collection phase of 10 to 19 months (32.4%, n = 12).

Twenty-nine studies considered consecutive admissions to GR units, without specifying age limitation, while sixteen studies included patients aged >65 years. Studies included patients with general mobility problems, post-orthopaedic interventions, stroke rehabilitation and rehabilitation following post-acute respiratory problems. However, they still focused on assessment measures of GR. Most excluded patients with medical instability (54.1%, n = 20) or with moderate-to-severe cognitive impairment (27.0%, n = 10). Two studies (5.4%) excluded participants from residential homes, while eight retrospective studies (21.6%) excluded participants with missing reported data.

### 3.3. Definition of Rehabilitation

Only a small number of the 37 selected studies provided a definition of rehabilitation, whereas most of them described other aspects of GR. The descriptions and definitions were heterogenous and lacked consensus.

Studies described the goal of rehabilitation as restoring functional independence and facilitating psychosocial adjustments to residual disability [9,13,14,15]. In most cases, a multidisciplinary team (MDT) established these objectives with the patient, and rehabilitation was deemed to be complete when the MDT determined that the goals had been achieved [16,17,18]. GR is qualitative and patient-specific. Nevertheless, studies regarded outcomes of rehabilitation as a yardstick for which to measure the degree of success or otherwise of the rehabilitation process [19,20]. These have been associated with the following: shorter length of stay; reduced mortality; return to previous home in the community; avoiding admission to nursing homes; and remaining out of hospital once discharged [2,4,21,22,23,24,25]. Patients were considered to have a higher RP if they were likely to be discharged back home to their previous residence in the community, following GR [26,27,28].

In some of the consulted studies, GR was affected by functional status, the presenting condition and comorbidities, age, cognition, social support and nutritional status [2,29,30]. Cognition also affected the level of compliance with rehabilitation and disability status [13,14,31,32]. GR success was also extended to post-inpatient discharge on further follow-up in the community [21,26].

### 3.4. Assessment for Geriatric Rehabilitation

The fact that GR primarily uses an MDT approach became evident upon noting which professional carried out the assessment. The highest percentage was completed by the MDT (40.5%, n = 15), while in some studies, the researchers performed the assessment (21.6%, n = 8) or collected data. In 18.9% of the studies (n = 7), it was not specified which clinician was involved. Others included physicians and geriatricians (5.4%, n = 2), occupational therapists (2.7%, n = 1), nurses (8.1%, n = 3) and physiotherapists (2.7%, n = 1). However, there were no details or explanations as to how the final decision of acceptance or rejection of inpatient GR was made. Although the clinicians were mentioned in most studies, it was unclear as to how or who rated RP affecting GR admission.

Some studies [3,14,23,33] evaluated patients’ self-reported goals and functional outcome measures in rehabilitation assessments. None of these, however, were the definitive criteria of acceptance for GR. Other studies also looked at clinician-evaluated RP compared with GR success, thus shedding light on how clinicians can rate potential, based on their clinical expertise [34,35].

The timings of the assessments that were carried out also varied. These were mostly performed at multiple time points, with more than half made at admission and discharge (54.1%, n = 20), whereas another 21.6% (n = 8) were carried out at admission, discharge and at follow-up post-discharge. Only five studies (13.5%) included comparator groups—that is, included a control group—for comparison between assessments and interventions.

### 3.5. Assessments Used for Geriatric Rehabilitation

In their respective assessments, the studies evaluated for this review measured rehabilitation differently from each other. This divergence proved to be very challenging in combining the findings in a systematic review or meta-analysis. The aims and hypotheses of the various studies were noted, and the main themes were extracted. The aims were mostly to evaluate which factors tend to impact rehabilitation. The studies looked at factors including frailty (8.1%, n = 3), cognition (13.5%, n = 5), pre-function (2.7%, n = 1), medical status (2.7%, n = 1) and patient goals (8.1%, n = 3). A total of 16.2% (n = 6) looked at factors impacting rehabilitation post-hip-fracture surgery. Some of the studies evaluated the use of specific outcome measures, while others used assessment tools to evaluate inpatient rehabilitation outcomes. Others investigated the relationships between function and mortality, age and rehabilitation and clinicians’ predictions of patient rehabilitation (Table 1).

The assessments included the various domains constituting the definition of rehabilitation endorsed by the WHO in its ICF framework, and other sub-domains that were extracted from the studies. The majority looked at patient demographics and characteristics (97.3%, n = 36), which included information such as gender, age, pre-admission living arrangements and settings. The most prevalent domains assessed for admission to GR are listed in Table 2.

A total of 172 outcome measures were used in the studies that met the criteria. Three of the studies each used a specific instrument to assess GR, namely, Minimum Data Set for Post-Acute Care (MDS-PAC) [9], Hospital Admission Risk Profile (HARP) [36] and gait, eyesight, mental state, sedation (GEMS) [29]. All three instruments employed a number of outcome measures from different domains, prior to having a final decision on GR potential. Table 3 portrays the highest-rated measure from each domain.

The majority of the remaining studies used more than one outcome measure from multiple domains during the assessment. Function was measured in most assessments (83.8%), followed by cognition (78.4%), with both domains being also commonly assessed in conjunction (67.6%). This summary of domain combinations is shown in Table S7.

All domains included other outcome measures. The cognition domain also included the Abbreviated Mental Test (AMT), which was used in three studies (8.1%); the Short Portable Mental Status Questionnaire (SPMSQ), which was employed in two studies (5.4%); and another 10 outcome measures that were used once (2.7% each).

Following the Barthel Index (BI) and the Functional Independence Measure (FIM) in most studies, three studies (8.1%) used a function-assessment sheet, designed by the clinicians in a hospital setting. However, no details of what the score sheet entailed were provided in the study report. Another 11 outcome measures were used once in different studies (2.7% each).

Medical assessments were used in 23 articles (62.2%) including comorbidities, number of medications and the admitting condition; once again, measures varied. Following the Charlson Comorbidity Index (CCI), the Cumulative Illness Rating Scale (CIRS) was used in four studies (10.8%) and nine other outcome measures were used once (2.7% each). The physical domain was assessed in 16 articles (43.2%). In none of these articles was it assessed on its own, but was combined with other domains. Six of these articles (16.2%) used a single outcome measure from the physical domain, with other outcome measures from other domains. The single outcome measures used were: POMA [32], the MDS-PAC instrument [9], WHODAS [3], the Short Physical Performance Battery test (SPPB) [37], the Hand-Grip Strength Test (HGS) [38] and the Timed Up and Go test (TUG) [4]. The remaining articles used multiple physical assessments. Following HGS and TUG, the SPPB and the minute-walk tests (MWT) were used in three studies (8.1%), and the Berg Balance Scale (BBS) was used in two studies (5.4%). A total of 10 other outcome measures were used once (2.7% each).

Thirteen articles (35.1%) investigated behaviour during assessment. Following the Geriatric Depression Scale (GDS), the Confusion Assessment Method (CAM) was used in three studies (8.1%), the Center for Epidemiologic Studies Depression Scale (CES-D) was used in two studies (5.4% each), and five other outcome measures were used once (2.7% each).

Various outcome measures were used to assess patient quality of life and its effect on GR (21.6%, n = 8). Two studies (5.4%) used a clinician-designed questionnaire based on ICF standards, but no specific information was provided in either study. Two further studies used the EQ-5D-3L, a questionnaire with a visual analogue scale (VAS) format, comprising questions of five domains being “mobility, self-care, usual activities, pain/discomfort and anxiety/depression” [39]. The remaining eight outcome measures were used in one study each, and varied between standardised measures and questionnaires, to provide an indication of the patient’s perceived emotional, social and functional state (2.7% each).

Four studies (10.8%) included nutrition in their assessment. These used the Mini Nutritional Assessment (5.4%, n = 2), the Malnutrition Screening Tool [37] and the Minimum Data Set for Post-Acute Care (MDS-PAC). The latter does not only assess nutrition, but is an instrument that assesses frailty, including domains of “cognition, communication/hearing, vision, mood and behaviour, social functioning, physical functioning (self-performance of ADLs and instrumental activities of daily living (IADLs), continence, disease diagnosis, health conditions, nutrition/hydration status, dental status, skin condition, and medications.” [9]. Another 18.9% (n = 7) of the studies measured frailty. Following CFS, the Frailty Index (FI) and the Fried Frailty Phenotype (FFP) were used in 2.7% of each [40,41].

Communication and vision assessments were carried out in two studies (5.4%), both of which formed part of an instrument that was being validated for the assessment of GR and RP, assessing multiple domains. These instruments were MDS-PAC and GEMS [9,29]. Another two outcome measures were used, assessing abuse, pain and nursing load (2.7%, n = 1 each) [26,37,42]. All these were used as part of a set of assessments.

The respective numbers of outcome measures employed for each domain are listed in Table 4. A detailed list of outcome measures used is shown in Tables S6–S16.

## 4. Discussion

Through this scoping review, the research team could establish that it is not yet possible to provide a watertight definition of GR and determine the factors that would condition RP. The diversity of the studies prevents a definitive answer at this time. There were significant discrepancies in the definition of rehabilitation and, in most cases, an absence of a clear definition was noted. A considerable number of different outcome measures and multiple domains were used (Table 4; Table S6). There was lacking evidence of consensus or a standard approach to GR assessment and potential scaling, but similar decision-making domains were used. The majority of studies sought to assess older adults in a holistic way, addressing the acute episode of ill health, and considering function, cognition, medical and psychosocial aspects of the patient (Table 2).

For successful GR, discharge planning should ideally start at the early stages of patient admission to hospital. This would help in developing a patient-specific plan of treatment, thus facilitating discharge and ensuring that the patient’s needs are met [6]. Assessing and identifying the functional abilities of daily living of patients—and comparing the level of functional ability prior to, and following, the acute onset—was given significant weight in the studies [41,43,44,45,46]. These were mostly assessed through the FIM or the BI, calculating the change in FIM total score at the initial stages of GR and the FIM score at discharge—or through a change in BI scores from admission to discharge. A positive change in these scores was considered as potential for GR. Other physical tests, in particular the TUG and the HGS, were used to assess the risk of falls and balance, hand-grip strength and frailty [29,40,41]. Slower or reduced improvement in functional ability could be due to cognitive impairment or behavioural problems. This was frequently assessed using the MMSE, thus addressing cognitive abilities that would influence RP and compliance with the programme. The GDS was used to assess mood and any disruptive behaviour that could impinge on rehabilitation compliance and performance. Patients’ perceptions, quality of life and motivation were also briefly mentioned using the EQ-5D-3L tool [34,37]. These tests were also a measure of evaluating the social support for patients and their environment.

The studies lacked standardised assessments of social support and environment. However, most pointed out that, in the case of patients residing in the community and enjoying a good level of support, it is reasonable to predict GR success. This also included the patient’s and carer’s wishes and rehabilitation goals [29,33,35]. Considering social factors is essential in discharge planning [6,26]. In the absence of support or caregivers who are able and willing to support the patient, a transition to a more supportive and monitored environment may be necessary. Conversely, should patients or caregivers opt for the patient to return home, then the necessary adaptations and provisions may be performed to facilitate a safe discharge home. This would require time and planning.

The studies illustrate the value of assessing comorbidities, as they are likely to affect GR success [2,30,37,47]. This was mostly assessed using the CCI, which made it possible to score the number of diseases and conditions of the patients under review. In the event of a high CCI score, there would be less potential for GR. Staff skill and clinical experience, and their impact on rehabilitation success were also explored [33,35]. It was maintained that clinical judgement and expectations were key factors in GR admission and the level of rehabilitation received.

The above-mentioned domains were in agreement with the Comprehensive Geriatric Assessment (CGA) and WHO ICF [8,10], which strongly points to the importance of functional and medical assessments in GR inpatient admission. The WHO ICF also states that spirituality and economic status also have a weighting in rehabilitation success, but none of the reviewed studies had these aspects in their assessments.

On the basis of the studies, the CGA and the WHO ICF, it is evident that using a single domain as an assessment of GR would fail to produce a correct prediction of rehabilitation success, and that a holistic approach would need to be adopted in addressing this complex field. Although there was no standardisation in the studies and no statistical significance could be noted through this review, it was apparent that function and cognition are the two main domains that tend to be assessed in conjunction for determining RP and have the greatest effect on rehabilitation success.

### Strengths and Limitations

This review followed a predetermined protocol and employed a thorough search technique [48,49]. The search strategy, in the opinion of the reviewers, was effective in discovering pertinent papers to be included in this review. However, some limitations were present. There is potential publication bias, as only research studies that were published in the English language were included. A common exclusion criterion in the studies was cognitive impairment, whereby patients with moderate or severe cognitive impairment were excluded from studies because it was deemed that such impairment could affect rehabilitation success and adherence to treatment programmes. This posed a further limitation, since patients with cognitive impairment are still commonly referred to GR and evidence suggests that patients with cognitive impairment could still benefit from GR input [23,32].

This review evaluated publications that were selected according to focused inclusion and exclusion criteria that were, in turn, aligned with a GR setting. Studies that included specialist rehabilitation or technology were excluded, due to specific specialist outcome measures and rehabilitation protocols. It is not to be excluded that outcome measures common to those studied in this review are also used in specialist settings. However, the linking of the GR setting and RP did not allow those publications to be included here.

The data presented by the various studies were summarised using quantitative measures, when possible. These tables and figures were intended to provide a summary, as the diversity of the studies was not conducive to undertaking an explanatory meta-analysis.

It proved to be a challenging task to identify studies that mentioned which outcome measures and assessment methods were used to assess RP and criteria for GR inpatient admission. This may be due to a limitation in the search terms and truncations used. However, it also sheds light on the lack of literature and consensus in GR decision-making and standardised frameworks available. Given the large body of literature on the subject, GR remains a challenging area to research and, while every attempt was made to identify the most pertinent studies, the presence of equally pertinent studies is not to be excluded.

## 5. Conclusions

This review did not set out to eliminate existing discrepancies in admission standards for inpatient geriatric rehabilitation (GR). However, it contributes to summarising the currently available research, highlighting the absence of standardised approaches and highlighting the necessity of achieving such standardisation.

Inpatient GR requires extensive resources and the involvement of multidisciplinary teams (MDT). This places a substantial expense on any healthcare system. The large number of outcome measures identified in this study highlights the complexities of this patient population. Moreover, the lack of consensus in standardised assessments for inpatient GR admission presents the MDT with a challenge in defining and understanding RP, and in accepting patients for GR.

Current assessments and approaches struggle to evaluate older adults in a holistic manner. They view certain domains of rehabilitation, such as cognition and physical function, but fail to consider others, thus also compromising inpatient GR success.

A standardised predictive model of rehabilitation, encompassing a holistic approach including outcome measures based on all the major domains, could have a positive impact on the GR admission system, and would allow an equitable chance for all older adults to be referred for inpatient GR. A standardised assessment process for GR could facilitate further research that would evaluate the effectiveness of GR in reducing hospital readmissions, decrease mortality rates and improve patient function. Further research is necessary towards managing this increasingly indispensable area of clinical practice.

## Figures and Tables

**Table 1:** The main themes extracted from the selected publications.

Theme of Study	Number of Articles	Percent of Articles (%)
Factors impacting rehabilitation	19	51.4
Specific outcome measures used for rehabilitation	11	29.7
Assessment of inpatient rehabilitation outcomes	6	16.2
Functional status and mortality after rehabilitation	5	13.5
Nurses’ predictions of rehabilitation	2	5.4
Outcomes for admission to rehabilitation	1	2.7
Rehabilitation applied to the oldest old	1	2.7

**Table 2:** Domains assessed for geriatric rehabilitation.

Domain	Number of Articles (n)	Percent out of Articles (%)
Function	31	83.8
Cognition	29	78.4
Medical	23	62.2
Physical	16	43.2
Behaviour	11	35.1
Quality of life	8	21.6
Frailty	7	18.9
Nutrition	4	10.8
Communication and vision	3	8.1
Abuse	1	2.7
Pain	1	2.7

**Table 3:** Most common measure from each domain for geriatric rehabilitation.

Outcome Measure	Outcome Measure Abbreviation	Number of Uses in Studies (n)	Percent out of Articles (%)
Mini Mental State Examination	MMSE	19	51.4
Barthel Index	BI	11	29.7
Functional Independence Measure	FIM	10	27.0
Charlson Comorbidity Index	CCI	10	27.0
Hand-Grip Strength Test	HGS	6	16.2
Timed Up and Go Test	TUG	6	16.2
Geriatric Depression Scale	GDS	6	16.2
Rockwood Clinical Frailty Score	CFS	6	16.2
Montreal Cognitive Assessment	MoCA	3	8.1
Clock Drawing and Clock Copying Test	CDT	3	8.1
Mini Nutritional Assessment	MNA	2	5.4
EQ-5D-3L	EQ-5D-3L	2	5.4

**Table 4:** Outcome measures per domain for geriatric rehabilitation.

Domain	Number of Outcome Measures (n)	Percent of Outcome Measures (%)
Cognition	40	23.3
Function	35	20.4
Physical	30	17.4
Medical	23	13.4
Behaviour	16	9.3
Quality of life	12	7.0
Frailty	8	4.7
Nutrition	4	2.3
Communication and vision	2	1.2
Abuse; Pain	2	1.2

## Data Availability

Not applicable.

## Supplementary Materials

The following supporting information can be downloaded at: https://www.mdpi.com/article/10.3390/jcm12030919/s1, Table S1: Search String; Table S2: Justification of Included Databases; Table S3: Article Data Extraction Form; Table S4: Sample of Excluded Articles; Table S5: Quality Component Affecters and Bias Assessment; Table S6: Domain combinations employed in the studies; Table S7: Outcome Measures from Domain 1: Function; Table S8: Outcome Measures from Domain 2: Cognition; Table S9: Outcome Measures from Domain 3: Medical; Table S10: Outcome Measures from Domain 4: Physical; Table S11: Outcome Measures from Domain 5: Nutrition; Table S12: Outcome Measures from Domain 6: Behaviour; Table S13: Outcome Measures from Domain 7: Quality of life; Table S14: Outcome Measures from Domain 8: Communication and vision; Table S15: Outcome Measures from Domain 9: Frailty; Table S16: Outcome Measures from Domain 10: Abuse, Pain; Table S17: Articles Chosen for the Review. References [50,51,52,53,54,55,56,57,58,59,60,61] are cited in the supplementary materials.

## Abbreviations

The outcome measures and tests used in this review are listed in Table S7, together with their abbreviations.

GR	Geriatric rehabilitation
MDT	Multidisciplinary team
RP	Rehabilitation potential
WHO	World Health Organization
ICF	International Classification of Functioning, Disability and Health
CGA	Comprehensive geriatric assessment
PADLs	Personal Activities of Daily Living
IADLs	Instrumental Activities of Daily Living
