# Supplementary material for: Assessment Tools for the Admission of Older Adults to Inpatient Rehabilitation: A Scoping Review
Source: J Clin Med. 2023 Jan 24;12(3):919. doi: 10.3390/jcm12030919 (PMC9918169; doi:10.3390/jcm12030919)
Supplement: Supplementary file 1 [file jcm-12-00919-s001.zip › jcm-2061529-supplementary.pdf]

Journal of
Clinical Medicine

MDPI

Table S1. Search String

Search	Search Terms
#1	(assess*) OR (estimate*) OR (evaluate*) OR (predict*)
#2	(geriatric*) OR (old*) OR (adult*) OR (age*) OR (elder*) OR (senior*)
#3	(admission) OR (access) OR (inpatient)
#4	rehab*
#5	(rehabilitation) AND (potential)
#6	(predict*) AND ((outcome) OR (score))
#7	#1 AND #2 AND #3 AND #4 AND #5 AND #6

is an indication that a different search line was used. The asterisk is a truncation, which is used to find word endings. e.g. assess* means the database will search assess, assesses, assessment, assessments etc.

Table S2. Justification of Included Databases

Database	Reason for Inclusion
ProQuest	ProQuest is a collection of many databases encompassing science journals and allied health sources, and is used by the University of Malta.
PEDro	PEDro gives rapid access to research, bibliographies, abstracts and evidence-based clinical practice guidelines in physiotherapy, including rehabilitation.
PubMed	PubMed contains bibliography lists from Medline, life science journals and online books. It provides links to other sources, with the aim of improving healthcare globally.
CINAHL Plus with full text (EBSCO)	CINAHL Plus with full text (EBSCO) contains indexing and bibliography lists and full text for nursing and allied health journals.
Cochrane Library	Cochrane Library is a collection of databases containing literature on healthcare decision-making. It contains many randomised controlled trials, which evaluate the effectiveness of healthcare interventions.

Table S3. Article Data Extraction Form

SCOPING REVIEW

ARTICLE DATA EXTRACTION

L-Università ta' Malta
Faculty of Health Sciences

Title of Study:
ASSESSMENT TOOLS FOR THE ADMISSION OF OLDER ADULTS TO INPATIENT REHABILITATION: A SCOPING REVIEW

Section 1: Article information

Author(s)	
Title	
Year	
Journal	

Section 2: Study setting

Type
Country
Place of study
Sample size
Timeline (months)

Section 3: Method

Aims/hypotheses
Inclusion criteria
Exclusion criteria
Domains assessed
Outcome measures used
Clinical follow-up
Comparator groups
Which clinician assessed

Section 4: Results

Main results
Limitations
Definition of rehabilitation

Table S4. Sample of Excluded Articles

Reason for Exclusion	Study
Participants <60 yrs	Konda et al., 2017 [50]
	Deom et al., 2021 [51]
	Zogg et al., 2020 [52]
Systematic reviews; focus groups	Cowley et al., 2021 [27]
	Sermon et al., 2021 [53]
	Lemos et al., 2021 [54]
Not specific to inpatient rehabilitation	Keppler et al., 2021 [55]
	Pai et al., 2021 [56]
	Pereira et al., 2011 [57]
Condition-specific	Belloni et al., 2021 [58]
	Kulzer et al., 2008 [59]
	McClure et al., 2011 [60]
Language	Munten et al., 2014 [61]

Table S5. Quality Component Affecters and Bias Assessment

	Myers et al., 2009 [35]	McPhail et al., 2013 [34]	Falahati et al., 2018 [3]	Vogt et al., 2008 [32]	Gillner et al., 1969 [42]	Calle et al., 2018 [44]	Muir-Hunter et al., 2016 [43]	Ruchinskas et al., 2001 [31]	Barnes et al., 2012 [47]	Smit et al., 2020 [14]	Aquilani et al., 2020 [24]	Low et al., 2020 [28]	Wan et al., 2021 [37]	Adunsky et al., 2003 [17]	Dutzi et al., 2019 [23]
Data collection timeline available	N	N	N	N	N	Y	Y	Y	Y	Y	Y	Y	Y	Y	Y
Definition of rehabilitation	N	N	N	N	Y	N	N	N	N	N	N	N	N	N	Y
Who carried out the assessment/researcher *	Y	Y	N	N	N	N	Y	Y	N	N	N	N	N	Y	N
Control group present	N	Y	N	N	Y	N	Y	Y	N	N	N	N	N	Y	N
Clear outcome measures stated **	Y	Y	Y	Y	N	Y	Y	Y	Y	Y	Y	Y	Y	Y	N

* studies incl. data collection carried out by researchers or not specified which clinician assessed. ** studies incl. clinician-designed outcome measures, no description given.

Table S6. Domain combinations employed in the studies

Combination	Count (n)	Per cent out of Articles (%)
Frailty	1	2.7
C	1	2.7
CP	1	2.7
CPM	1	2.7
CPMB	1	2.7
F	2	5.4
FC	5	13.5
FCM	5	13.5
FCMB	3	8.1
FCPB	3	8.1
FCPM	5	13.5
FCPMB	4	10.8
FM	3	8.1
FP	1	2.7
M	1	2.7

* F = function; C = cognition; M = medical; P = physical; B = behaviour. ** F = 83.8%; C = 78.4%; M = 62.2%; P = 43.2%; B = 29.7%.

Table S7. Outcome Measures from Domain 1: Function

Outcome Measure	Abbreviation	Responses (n)	Per cent out of Articles (%)
Barthel Index	BI	11	29.7
Functional Independence Measure	FIM	10	27.0
Clinician ADL Score Sheet	--	3	8.1
Functional Autonomy Measurement System	SMAF	1	2.7
Minimum DataSet for Post-Acute Care	MDS-PAC	1	2.7
Goal-Attainment Scaling	GAS	1	2.7
Lawton and Brody Scale	LBS	1	2.7
Sunnaas ADL Index	SI	1	2.7
Hospital Admission Risk Profile	HARP	1	2.7
Montebello Rehabilitation Factor Score	MRFS	1	2.7
Katz ADL Index	Katz ADL	1	2.7
Patient-Reported Outcomes Measurement Information System—Physical Function—Geriatric Rehabilitation	PROMIS-PF-GR	1	2.7
Older Americans' Resources and Services Functional Assessment	OARS	1	2.7
World Health Organization Disability Assessment Schedule	WHODAS	1	2.7

Table S8. Outcome Measures from Domain 2: Cognition

Outcome Measure	Abbreviation	Responses (n)	Per cent out of Articles (%)
Mini Mental State Examination	MMSE	19	51.4
Clock Drawing and Clock Copying Test	CDT	3	8.1
Montreal Cognitive Assessment	MoCA	3	8.1
Abbreviated Mental Test	AMT	3	8.1

Pfeiffer Short Portable Mental Status Questionnaire	SPMSQ	2	5.4
Mini-Cog Scale	--	1	2.7
Rowland University Dementia Assessment Scale	RUDAS	1	2.7
Gait, eyesight, mental state, sedation	GEMS	1	2.7
Minimum DataSet for Post-Acute Care	MDS-PAC	1	2.7
Red Cross Hospital Mental Scale	RCHMS	1	2.7
Neurobehavioral Status Cognitive Screening Examination	NCSE	1	2.7
Mattis Dementia Rating Scale	DRS	1	2.7
Hospital Admission Risk Profile	HARP	1	2.7
Trail Making Test	Trails	1	2.7
Motor-Free Visual Perception Test	MVPT	1	2.7

Table S9. Outcome Measures from Domain 3: Medical

Outcome Measure	Abbreviation	Responses (n)	Per cent out of Articles (%)
Charlson Comorbidity Index	CCI	10	27.0
Cumulative Illness Rating Scale	CIRS	4	10.8
Clinical Neuro Testing	--	1	2.7
Minimum DataSet for Post-Acute Care	MDS-PAC	1	2.7
GOLD Comorbidity Classification	--	1	2.7
Fine Prognostic Score	FPS	1	2.7
Canadian Neurological Scale	CNS	1	2.7
Hospital Admission Risk Profile	HARP	1	2.7
Weighted Functional Comorbidity Index	wFCI	1	2.7
Vulnerable Elders Survey	VES	1	2.7
ergebnisorientiertes Pflege Assessment Acute Care	ePA-AC	1	2.7

Table S10. Outcome Measures from Domain 4: Physical

Outcome Measure	Abbreviation	Responses (n)	Per cent out of Articles (%)
Hand Grip Strength Test	HGS	6	16.2
Timed Up and Go Test	TUG	6	16.2
Short Physical Performance Battery	SPPB	3	8.1
Minute-Walk Test	MWT	3	8.1
Berg Balance Scale	BBS	2	5.4
Gait, eyesight, mental state, sedation	GEMS	1	2.7
180° Turn Test	--	1	2.7
5 Sit-To-Stand Test	5STS	1	2.7
Gait Rite	--	1	2.7
Clinical range-of-motion testing	ROM	1	2.7
Minimum DataSet for Post-Acute Care	MDS-PAC	1	2.7
1RM Measure	1RM	1	2.7
Elderly Mobility Scale	EMS	1	2.7
Tinetti's Performance-Oriented Mobility Assessment	POMA	1	2.7
Hierarchical Assessment of Balance and Mobility	HABAM	1	2.7

Table S11. Outcome Measures from Domain 5: Nutrition

Outcome Measure	Abbreviation	Responses (n)	Per cent out of Articles (%)
Mini Nutritional Assessment	MNA	2	5.4
Malnutrition Screening Tool	MST	1	2.7
Minimum DataSet for Post-Acute Care	MDS-PAC	1	2.7

Table S12. Outcome Measures from Domain 6: Behaviour

Outcome Measure	Abbreviation	Responses (n)	Per cent out of Articles (%)
Geriatric Depression Scale	GDS	6	16.2
Confusion Assessment Measure	CAM	3	8.1
Center for Epidemiologic Studies Depression Scale	CES-D	2	5.4
Minimum DataSet for Post-Acute Care	MDS-PAC	1	2.7
Hospital Anxiety and Depression Scale	HADS	1	2.7
Behavioral Dyscontrol Scale	BDS	1	2.7
Delirium Observational Score	DOS	1	2.7
General Well-being Schedule	GWBS	1	2.7

Table S13. Outcome Measures from Domain 7: Quality of life

Outcome Measure	Abbreviation	Responses (n)	Per cent out of Articles (%)
EQ-5D-3L	EQ-5D-3L	2	5.4
Questionnaires based on ICF	--	2	5.4
Self-rated health questionnaire	SRHQ	1	2.7
Admission/discharge patient survey	--	1	2.7
Umea Life Satisfaction Checklist	LSC	1	2.7
Symptom/Emotion Checklist-10	SCL-10	1	2.7
Hospital Admission Risk Profile	HARP	1	2.7
Ageing Calculator	--	1	2.7
Nottingham Health Profile	NHP	1	2.7
Clinician questionnaire	--	1	2.7

Table S14. Outcome Measures from Domain 8: Communication and vision

Outcome Measure	Abbreviation	Responses (n)	Per cent out of Articles (%)
Minimum DataSet for Post-Acute Care	MDS-PAC	1	2.7
Gait, eyesight, mental state, sedation	GEMS	1	2.7

Table S15. Outcome Measures from Domain 9: Frailty

Outcome Measure	Abbreviation	Responses (n)	Per cent out of Articles (%)
Rockwood Clinical Frailty Score	CFS	6	16.2
Frailty Index	FI	1	2.7
Fried Frailty Phenotype	FFP	1	2.7

Table S16. Outcome Measures from Domain 10: Abuse, Pain

Outcome Measure	Abbreviation	Responses (n)	Per cent out of Articles (%)

Abuse screen	--	1	2.7
Visual analogue scale (pain)	VAS	1	2.7

Table S17. Articles Chosen for the Review

Articles chosen for the review, showing domains assessed with outcome measures for geriatric rehabilitation

Ref.	Article Information	Domains Assessed with Outcome Measures	Outcome Measures Used
[2]	Elphick, et al. (2007)	cognition, function	AMT, BI
[3]	Falahati, et al. (2018)	cognition, function, behaviour, physical	OARS, GDS, WHODAS
[4]	Kool, et al. (2017)	cognition, behaviour, physical, medical	TUG, GDS, MMSE, CIRS, VES, ePA-AC
[9]	Landi, et al. (2002)	cognition, communication, function, physical, behaviour, nutrition, medical	MDS-PAC
[13]	Hershkovitz, et al. (2007)	cognition, function	MMSE, FIM
[14]	Smit, et al. (2020)	cognition, function, medical	CCI, MMSE, PROMIS-PF-GR
[15]	Kabboord, et al. (2020)	medical, physical, function, cognition	CCI, wFCI, EMS, BI, MoCA
[16]	Adunsky, et al.(2001)	function	FIM
[17]	Adunsky, et al. (2003)	function, cognition	FIM, MRFS
[18]	Baztan, et al. (2009).	function, medical, cognition	BI, CCI, SPMSQ
[19]	D'Alton, et al. (2019)	frailty	CFS
[20]	Volpato, et al. (2008)	physical, cognition, function, behaviour, medical	SPPB, HGS, LBS, MMSE, CES-D, CIRS, Gold, FPS, CNS
[21]	Chin, et al. (2008)	cognition, function	AMT, FIM
[22]	Sweet, et al. (2011)	function, cognition, medical, behaviour	FIM, MMSE, MoCA, CIRS, GDS
[23]	Dutzi, et al. (2019)	behaviour, frailty, cognition, function, QOL, physical	GDS, CFS, MMSE, BI, ICF questionnaire, 5STS, 1RM, GaitRite, TUG, HABAM, POMA
[24]	Aquilani, et al. (2021)	function	BI
[25]	Stolee, et al. (1999)	function, cognition, QOL	BI, MMSE, QOL, SRHQ, GAS
[26]	Gosselin, et al. (2008)	medical, cognition, pain, physical, function	CCI, MMSE, Trails, MVPT, VAS, BBS, TUG, HGS, GWBS, SMAF
[28]	Low, et al. (2020)	medical, function, frailty	CCI, CFS, FIM
[29]	Jupp, et al. (2011)	physical, cognition, medical, communication	TUG, MMSE, AMT, GEMS
[30]	Semel, et al. (2010)	function	FIM
[31]	Ruchinskas, et al. (2001)	cognition, functional, medical, behaviour	CDT, NCSE, DRS, FIM, GDS
[32]	Vogt, et al. (2008)	cognition, physical	MMSE, POMA
[33]	Kus, et al. (2011)	function, QOL	questionnaire function and QOL, ICF classification
[34]	McPhail, et al. (2013)	QOL, cognition	MMSE, EQ-5D-3L
[35]	Myers, et al. (2009)	medical, QOL, function	CCI, BI, Admission/Discharge Patient Survey, BDS
[36]	Liu, et al. (2016)	cognition, QOL, medical, function	HARP, MMSE
[37]	Wan, et al. (2021)	QOL, function, abuse, nutrition, frailty, medical, physical, cognition, behaviour	CFS, ADLs, SPPB, EQ-5D-3L, CCI, CIRS, MMSE, MoCA, RUDAS, GDS, abuse screen, MST
[38]	Nouvenne, et al. (2021)	medical, physical, function, frailty, behaviour, cognition	HGS, Katz ADL, CFS, CAM, mini-COG
[40]	Troster, et al. (2020)	function, physical, frailty, nutrition, cognition, medical	BI, SPPB, FFP, HGS, MMSE, CDT, MNA, DOS
[41]	Singh, et al. (2012)	medical, frailty, function, physical, cognition	CCI, FI, BI, MMSE, CDT, FIM, HGS, 180 turn, TUG
[42]	Gillner, et al. (1969)	physical	MWT
[43]	Muir-Hunter, et al.	physical, cognition, function, behaviour	FIM, MMSE, GDS, BBS, TUG, 2MWT

	(2016)		
[44]	Calle, et al. (2018)	function, cognition, physical, medical, nutrition	CCI, MNA, MMSE, CAM, HGS, 4MWT, BI
[45]	Johansen, et al. (2011)	function, QOL, cognition	SI, LSC, MMSE, SCL-10
[46]	Valderrama-Gama, et al. (1998)	function, cognition	BI, RCHMS
[47]	Barnes, et al. (2012)	medical, behaviour, cognition, function	Ageing Calculator, CCI, CES-D, SPMSQ
